# Evaluation of the global lung function initiative 2012 reference values for spirometry in a Swedish population sample

**DOI:** 10.1186/s12890-015-0022-2

**Published:** 2015-03-25

**Authors:** Helena Backman, Anne Lindberg, Anssi Sovijärvi, Kjell Larsson, Bo Lundbäck, Eva Rönmark

**Affiliations:** Department of Public Health and Clinical Medicine, Division of Occupational and Environmental Medicine, the OLIN unit, Umeå university, Umeå, Sweden; Department of Public Health and Clinical Medicine, Division of Medicine, the OLIN unit, Umeå University, Umeå, Sweden; Department of Clinical Physiology and Nuclear Medicine, HUS Medical Imaging Centre, Helsinki University Central Hospital, Helsinki, Finland; Division of Physiology, the National Institute of Environmental Medicine, Karolinska Institutet, Stockholm, Sweden; Krefting Research Centre, Institute of Medicine, University of Gothenburg, Gothenburg, Sweden; OLIN-Studierna, Robertsviksgatan 9, 97189 Lulea, Sweden

**Keywords:** Lung function, Spirometry, Reference values, Z-score, Lower Limit of Normal

## Abstract

**Background:**

The Global Lung Function Initiative 2012 (GLI) reference values are currently endorsed by several respiratory societies but evaluations of applicability for adults resident in European countries are lacking. The aim of this study was to evaluate if the GLI reference values are appropriate for an adult Caucasian Swedish population.

**Methods:**

During 2008–2013, clinical examinations including spirometry were performed on general population samples in northern Sweden, in which 501 healthy Caucasian non-smokers were identified. Predicted GLI reference values and Z-scores were calculated for each healthy non-smoking subject and the distributions and mean values for FEV_1_, FVC and the FEV_1_/FVC ratio were examined. The prevalence of airway obstruction among these healthy non-smokers was calculated based on the Lower Limit of normal (LLN) criterion (lower fifth percentile) for the FEV_1_/FVC ratio. Thus, by definition, a prevalence of 5% was expected.

**Results:**

The Z-scores for FEV_1_, FVC and FEV_1_/FVC were reasonably, although not perfectly, normally distributed, but not centred on zero. Both predicted FEV_1_ and, in particular, FVC were lower compared to the observed values in the sample. The deviations were greater among women compared to men. The prevalence of airway obstruction based on the LLN criterion for the FEV_1_/FVC ratio was 9.4% among women and 2.7% among men.

**Conclusions:**

The use of the GLI reference values may produce biased prevalence estimates of airway obstruction in Sweden, especially among women. These results demonstrate the importance of validating the GLI reference values in different countries.

**Electronic supplementary material:**

The online version of this article (doi:10.1186/s12890-015-0022-2) contains supplementary material, which is available to authorized users.

## Background

Reference values for spirometry are necessary for identifying subjects with abnormal lung function. The European Coal and Steel Community (ECSC) reference values [1] have until recently been recommended for European countries by the European Respiratory Society (ERS). In Sweden, two domestic reference values have been widely used [2-4]. In 2012, The Global Lung Function Initiative (GLI), an ERS task force, presented new multi-ethnic reference values for spirometry [5] for several different ethnicities within the three to 95 years age-span. These GLI reference values are currently endorsed by several respiratory societies [5,6]. For Caucasians, the GLI reference values are based on data from asymptomatic lifelong non-smokers from 30 different centres comprising 57,395 subjects with European ancestry from several European countries including Sweden, along with Israel, Australia, USA, Canada, Brazil, Chile, Mexico, Uruguay, Venezuela, Algeria and Tunisia. They have been evaluated and found to be applicable for the Australasian population aged 4–80 years [7] as well as for British children [8], but do not reflect data for Tunisian adults very well [9]. Further evaluations of applicability from other parts of the world are required in order to verify the appropriateness in these areas. Hitherto, there are no publications evaluating the applicability of the GLI reference values for Caucasian adult residents in any of the European countries.

The definition of airway obstruction is based on the ratio between forced expiratory volume in one second (FEV_1_) and the vital capacity (VC) measured by slow (SVC) and/or forced (FVC) manoeuvres. According to the Global Initiative on Obstructive Lung Disease (GOLD) a post-bronchodilator ratio < 0.7 is defined as not fully reversible airway obstruction [10]. The ERS and American Thoracic Society (ATS) recommend the use of Lower Limit of Normal (LLN) defined as the lower fifth percentile of the distribution for healthy non-smokers to define an abnormally low ratio [11-13]. The LLN definition is dependent on the set of reference values in use. Consequently, when implementing the LLN criteria in a population of healthy non-smoking subjects, a prevalence of obstruction of 5% indicates perfect applicability of the reference values in use.

It is of great importance that the population from which the reference values are derived is representative for the population under study. The age distribution and other anthropometric, ethnic, environmental and socio-economic factors should be equivalent since such factors can affect lung function. Additionally, the methodology for performing spirometric measurements in terms of protocol and equipment etc. must be stringent [11,14].

The aim of the present study was to evaluate if the GLI reference values, although endorsed by several respiratory societies including the ERS and ATS, are applicable for an adult Caucasian population resident in Sweden.

## Methods

### Study design and reference population

The study sample was recruited from the Obstructive Lung Disease in Northern Sweden (OLIN) Studies population-based cohorts. In 2006, a randomly selected cohort in ages 20–69 years was invited to a postal questionnaire survey (n = 7,997) together with a follow-up of a previously recruited randomly selected cohort aged 30–84 years in 2006 (n = 7,004). Of the responders (n = 12,055, 80.4%), 1016 subjects were randomly selected after stratification reflecting the age and sex distribution of the general population in the study area. They were invited to examinations including structured interviews and lung function tests in which 726 subjects (71.5%) participated. In order to obtain a sufficient number of healthy non-smokers, an additional sample of 738 healthy non-smokers according to the 2006 questionnaire survey were also invited to the examinations and 448 (60.7%) participated. All examinations were performed during 2008–2013, and, in total, 501 Caucasians (49% women) were identified as healthy non-smokers with acceptable spirometry quality and constitute the reference population.

Healthy non-smokers [15] were defined as subjects without a history of any airway or lung disease, breathlessness, cough, wheeze, ischemic heart disease, rheumatic disorders or a previous life-time exposure of > one pack-year of smoking. Their characteristics are described in Table 1. Eligibility criteria are presented in an appendix [see Additional file 1]. The age was calculated by one decimal point as the difference between date of birth and date of examination. Height was measured in stocking feet with an accurate stadiometer with 0.5 cm precision. Weight was measured with 0.5 kg precision without jacket and shoes and with empty pockets. Written informed consent for participation in the study was obtained from the participants and the study was approved by the Regional Ethical Review Board at Umeå University, Sweden.

**Table 1:** **Characteristics of the reference population**

	**Women 244**	**Men 257**
Mean age ± SD (range) in years	49.2 ± 17.6 (22.6-91.3)	46.6 ± 15.9 (22.7-86.7)
Mean height ± SD (range) in cm	163.3 ± 6.7 (139–181)	178.9 ± 7.0 (162–198)
Mean weight ± SD (range) in kg	68.2 ± 12.1 (45.0-118.0)	84.7 ± 13.5 (56.0-148.0)
Mean BMI ± SD (range)	25.6 ± 4.2 (17.1-39.0)	26.4 ± 3.7 (18.9-44.1)
Mean FEV_1_ ± SD	2.88 ± 0.67	4.18 ± 0.78
Mean FVC ± SD	3.66 ± 0.81	5.30 ± 0.94
Mean SVC ± SD	3.71 ± 0.82	5.43 ± 0.94
Mean VC ± SD	3.73 ± 0.81	5.45 ± 0.94
Mean FEV_1_/FVC ± SD	0.785 ± 0.064	0.788 ± 0.056
Mean FEV_1_/VC ± SD	0.770 ± 0.067	0.766 ± 0.060

N = Number of subjects, SD = Standard Deviation, BMI = Body Mass Index, FEV_1_ = Forced Expiratory Volume in one second, FVC = Forced Expiratory Vital Capacity, SVC = Slow Expiratory Vital Capacity, VC = Vital Capacity; the highest value of FVC and SVC. All variables except for age and the FEV_1_/FVC and FEV_1_/VC ratios differed significantly between men and women.

### Spirometric measurements

Spirometric measurements included FEV_1_, FVC and SVC performed on two Jaeger Masterscope spirometers (JLAB version 5.21, CareFusion, Würzburg, Germany). The spirometers were calibrated each morning, and a minimum of three and a maximum of eight measurements were performed per subject. The procedures were performed without use of bronchodilators and following the ATS/ERS recommendations [16] but with a repeatability criterion of ≤5% deviation from the second highest value [17]. Once the data had been exported from the spirometers to an electronic data file, data validity controls were performed. The repeatability criterion was not met for 2% of the measurements and each such measurement was thoroughly examined by post-hoc ocular control of flow-volume charts, resulting in the exclusion of two subjects.

### Statistical analysis

The GLI reference values are based on pre-bronchodilator values, and only pre-bronchodilator values from the reference population were collected and analysed. Using the Excel macro for GLI [18], reference values, lower limit of normal (LLN), Z-scores and percentiles for FEV_1_, FVC and the FEV_1_/FVC ratio were calculated for each subject in the reference population. The GLI Z-score is a standardized measure of the positioning of an observed measurement in the distribution of the population from which the GLI reference values are derived and takes both between-subject and age- and height-related variability into account. If the agreement between the observed values in the reference population and the GLI reference values is perfect, the mean Z-scores should ideally be zero, and the standard deviation (SD) should be one [19]. Mean values and standard deviations were calculated, and Q-Q plots were scrutinized to determine if the Z-scores were normally distributed. Possible relationships between Z-scores and age, height, weight and sex were examined by multiple linear regression models. If the GLI reference values are applicable, no such relationship should exist. LLN was defined as the lower fifth percentile in the distribution from which the GLI reference values are derived, as calculated by the GLI Excel macro, if not explicitly stated otherwise. The 90% limits of normality, which are expected to include 90% of the observations if the agreement is perfect, were defined as observations with GLI Z-scores within the −1.645 to +1.645 ranges. To identify the lower 2.5th percentile, the Z-score threshold of −1.96 was used.

For comparison, a number of other commonly used reference values were also applied on the reference population, i.e. ECSC [1] and Hankinson [20], and from Scandinavia, Berglund [2], Hedenström [3,4], Langhammer [21], Gulsvik [22] and Viljanen [23]. Mean observed measurements, expressed as percent of predicted, were calculated across the different sets of reference values.

## Results

### The distribution of GLI Z-scores

**Figure 1:**
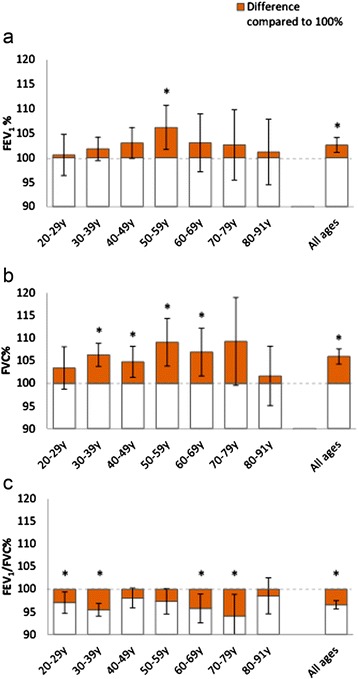
**Mean observed values of a) FEV1, b) FVC and c) FEV1/FVC in percent of GLI reference values by age group, among women.** 95% Confidence Intervals for the difference between mean% and 100% are displayed. An asterix (*) denotes p<0.05 for independent samples T-test of difference compared to 100%.

**Figure 2:**
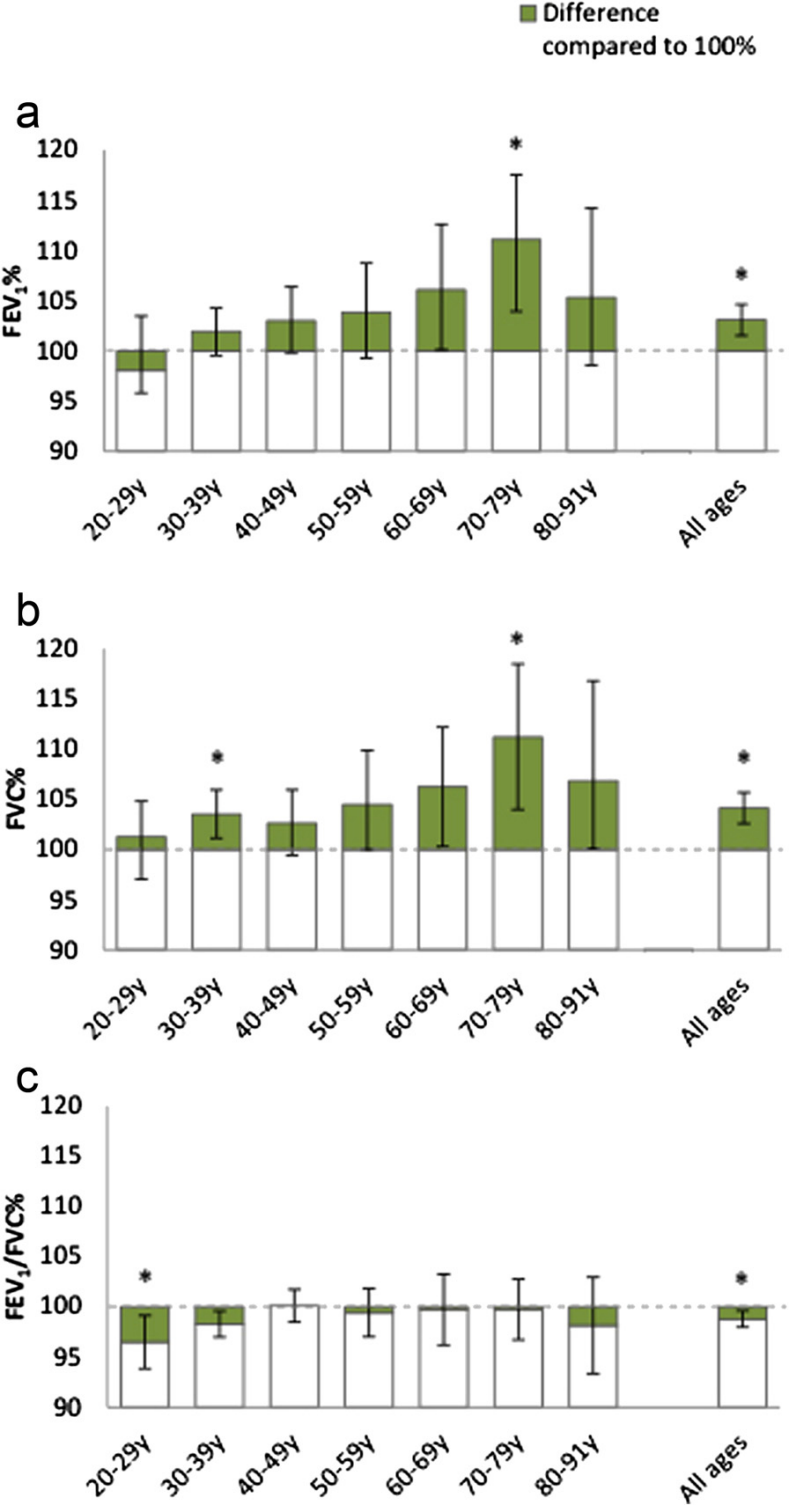
**Mean observed values of a) FEV1, b) FVC and c) FEV1/FVC in percent of GLI reference values by age group, among men.** 95% Confidence Intervals for the difference between mean% and 100% are displayed. An asterix (*) denotes p<0.05 for independent samples T-test of difference compared to 100%.

When applying the GLI reference values [5] on the reference population, the Z-scores for FEV_1_, FVC and FEV_1_/FVC were reasonably, although not perfectly, normally distributed, but not centred around zero. The mean Z-score was 0.21 (SD 0.91) for FEV_1_, 0.35 (SD 0.92) for FVC and −0.25 (SD 0.85) for the FEV_1_/FVC ratio and differed significantly from zero for all three measures (p < 0.001). Both FEV_1_ and FVC exceeded the predicted values across all ages except for FEV_1_ among 22–29 year olds. The pattern was the opposite for the FEV_1_/FVC ratio, where Z-scores generally were below zero across all ages, in particular among women. Independent samples T-tests showed a significant difference in mean Z-scores between women and men for the FEV_1_/FVC ratio, but not for FEV_1_ or FVC [Table 2]. Mean Z-scores deviated significantly from zero in the same age groups as where mean percent of predicted significantly deviated from 100% as displayed in Figures 1 and 2.

**Table 2:** **Mean GLI Z-scores for FEV**_**1**_ **, FVC and the FEV**_**1**_ **/FVC ratio by age group and sex**

			**FEV**_**1**_			**FVC**			**FEV**_**1**_ **/FVC**		
**Age group**	**Sex**	**N**	**Mean**	**(SD)**	**p-value**	**Mean**	**(SD)**	**p-value**	**Mean**	**(SD)**	**p-value**
22-29 y	Women	28	0.05	(0.92)		0.26	(0.97)		−0.35	(0.83)	
	Men	32	−0.16	(0.80)	0.346	0.11	(0.84)	0.509	−0.41	(0.97)	0.818
30-39 y	Women	78	0.16	(0.89)		0.49	(0.89)		**−0.56**	**(0.80)**	
	Men	86	0.17	(0.88)	0.937	0.28	(0.92)	0.143	**−0.20**	**(0.81)**	**0.006**
40-49 y	Women	39	0.25	(0.78)		0.36	(0.82)		−0.21	(0.89)	
	Men	53	0.25	(0.98)	0.999	0.20	(0.95)	0.381	0.04	(0.80)	0.152
50-59 y	Women	35	0.46	(0.96)		0.61	(1.05)		−0.27	(0.98)	
	Men	31	0.29	(0.98)	0.464	0.31	(1.04)	0.245	−0.04	(0.80)	0.310
60-69 y	Women	21	0.20	(0.86)		0.43	(0.71)		−0.44	(0.74)	
	Men	24	0.41	(1.04)	0.481	0.41	(0.93)	0.939	−0.01	(0.88)	0.080
70-79 y	Women	19	0.16	(0.88)		0.51	(1.12)		−0.54	(0.94)	
	Men	17	0.68	(0.77)	0.071	0.68	(0.87)	0.604	−0.02	(0.57)	0.051
80-91 y	Women	24	0.07	(0.85)		0.07	(0.80)		−0.10	(0.86)	
	Men	14	0.30	(0.85)	0.420	0.38	(0.96)	0.292	−0.14	(0.68)	0.884
<40 y	Women	106	0.13	(0.90)		0.43	(0.91)		**−0.50**	**(0.81)**	
	Men	118	0.08	(0.87)	0.671	0.24	(0.90)	0.108	**−0.26**	**(0.86)**	**0.030**
≥40 y	Women	138	0.25	(0.86)		0.41	(0.92)		**−0.29**	**(0.89)**	
	Men	139	0.34	(0.95)	0.415	0.34	(0.96)	0.542	**−0.01**	**(0.77)**	**0.006**
All ages	Women	244	0.20	(0.88)		0.42	(0.91)		**−0.38**	**(0.86)**	
	Men	257	0.22	(0.92)	0.784	0.29	(0.93)	0.126	**−0.12**	**(0.82)**	**0.001**

SD = standard deviation.

P-values for difference between sexes (independent samples *T*-test).

Bolded figures indicate p-values < 0.05 for difference between sexes.

GLI = Global Lung Function Initiative.

FEV_1_ = Forced Expiratory Volume in one second.

FVC = Forced Expiratory Vital Capacity.

**Figure 3:**
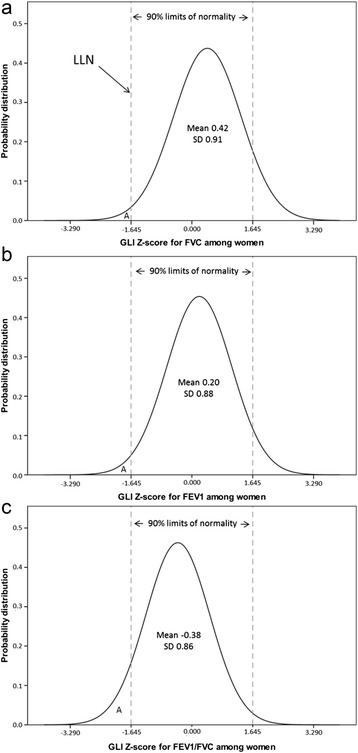
**Normal distribution curves of a) FVC, b) FEV1 and c) FEV1/FVC based on observed GLI Z-score means and standard deviations among women.** The figures illustrate observed values of Z-score mean and standard deviations (SD) among women. LLN=Lower Limit of Normal. X=proportion of subjects with values below LLN. Observed values of X are 0.4% for FEV1 (n=1), 0.8% for FVC (n=2) and 9.4% for FEV1/FVC (n=23).

For FEV_1_, 93.6% (94.3% among women, 93.0% among men) of the 501 observations were within the 90% limits of normality. For FVC, the corresponding proportion was 90.2% (89.3% among women, 91.1% among men), and for the FEV_1_/FVC ratio, the proportion was 92.2% (88.5% among women, 95.7% among men). However, only 2.0% of the women and 1.6% of the men had FEV_1_/FVC values above the 95th percentile. The pattern of Z-scores was the opposite for FEV_1_ and FVC. For FEV_1_, 0.4% of the women and 1.2% of the men had values below the 5th percentile, and 5.3% of the women and 5.8% of the men above. For FVC, 0.8% of the women and 1.2% of the men had values below the fifth percentile, and 9.8% of the women and 7.8% of the men above. The GLI percentile frequency distributions for FEV_1_, FVC and the FEV_1_/FVC ratio were shifted, particularly among women, towards higher percentiles for FEV_1_ and FVC, and towards lower percentiles for FEV_1_/FVC [Figure 3].

### Factors related to GLI Z-scores

When analysing the Z-scores for FEV_1_, FVC and the FEV_1_/FVC ratio in relation to age, height, weight and sex, small but statistically significant associations yielding R-square values of 1-2% were found. Sex had a significant impact on the Z-score for the FEV_1_/FVC ratio (Beta-coefficient 0.256 (p = 0.001)), sex and height had a significant impact on the Z-score for FEV_1_ (Beta-coefficient 0.276 (p = 0.023) for sex, −0.016 (p = 0.005) for height), and weight had a significant impact on the FVC Z-score (Beta-coefficient −0.009 (p = 0.001)).

### Prevalence of airway obstruction according to LLN based on GLI reference values

**Figure 4:**
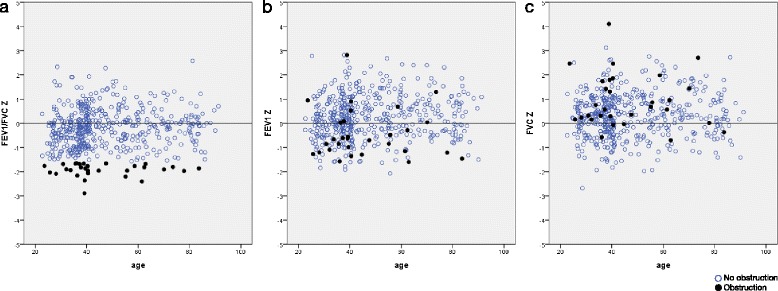
**GLI Z-scores for a) FEV1/FVC, b) FEV1 and c) FVC among healthy non-smoking subjects with and without airway obstruction, by age.** Airway obstruction was defined according to the Lower Limit of Normal criterion (below 5th percentile) for the FEV1/FVC ratio based on the GLI reference values.

The prevalence of spirometrically defined airway obstruction in the reference population according to LLN for the FEV_1_/FVC ratio was 9.4% (95% CI 5.7%-13.1%) among women and 2.7% (95% CI 0.7%-4.7%) among men (p-value = 0.002). When applying the lower 2.5th GLI percentile as LLN; the prevalence of obstruction in the reference population was 3.4% (95% CI 1.1%-5.7%) among women and 1.5% (95% CI 0.0%-3.0%) among men. Figure 4. illustrates that subjects defined as having obstruction according to the LLN criterion tended to have Z-scores for FEV_1_ below rather than above zero and Z-scores for FVC above rather than below zero.

### Comparison to other reference values

**Figure 5:**
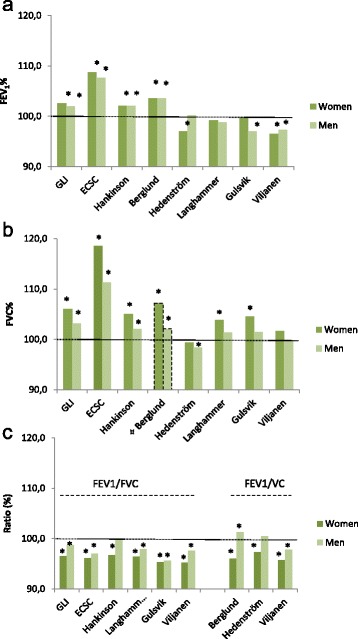
**Mean values of a) FEV1, b) FVC and c) the Ratio expressed as percent of predicted according to different reference values, by sex.** Mean percent of predicted values are displayed for FEV1, FVC and the FEV1/FVC and FEV1/VC ratios based on data from a population-based sample of healthy non-smoking subjects. The age-span included is 22 to 65 years (n=192 women and n=218 men) in which all reference values can be applied without extrapolation. GLI=Global Lung Initiative 2012, ECSC=European Coal and Steel Community. *p-value<0.05 (one-sample T-test compared to 100%). ¤ Berglund reference values for FVC represent VC; i.e. the best of FVC and SVC.

When other sets of reference values were applied to the reference population, the mean FEV_1_ and FVC as percent of predicted significantly exceed 100% for both sexes according to ECSC, Hankinson and Berglund. Mean FEV_1_ and FVC as percent of predicted for reference values Hedenström (Sweden), Langhammer (Norway), Gulsvik (Norway) and Viljanen (Finland) were closer to 100%. In general, the FVC percent of predicted values were more overestimated than the FEV_1_ values for all sets of reference values, and particularly so among women. Consequently, FEV_1_/FVC and FEV_1_/VC as percent of predicted were generally below 100%, and more pronounced so among women compared to men [Figure 5].

## Discussion

Compared to the ECSC reference values, the GLI reference values are superior, but not perfect, for Swedish adults. The original intention of GLI was that the same reference values should be possible to use in most parts of the world, covering different ethnicities and ages to avoid age-related junction points between different sets of reference values. The data which the GLI equations are based on were collected from 1978 to 2008, which may question whether or not the oldest data still are valid. However, earlier studies by Quanjer et al. found no evidence of impact of secular trends in FEV_1_, FVC or FEV_1_/FVC in Caucasians during the last 30 years. They also found that reference equations derived from collated datasets, such as the GLI, are applicable across different centres using different equipment, which is another strong argument for using GLI [24].

The GLI reference values represent the average of all available data they are based on and may thus not be representative for every specific subpopulation included. Since there are substantial differences in e.g. occupational exposures and environmental pollution which may affect lung function between countries and regions populated by Caucasians, differences in lung function can be expected. Data from Swedish centres are included in the reference data from which the GLI reference values are derived, but comprise only 123 subjects. Since there are substantial differences in anthropometric, environmental and socio-economic factors between e.g. Scandinavia and southern Europe, an evaluation of the fit for Swedish subjects is required.

Swanney et al. [25] argues that adopting the GLI reference values in clinical practice worldwide is essential and urgent, in order to reduce the confusion regarding which reference values to rely on. In essence, Swanney et al. argues that the use of GLI worldwide is preferable to local specific reference values obtained with different techniques, especially since the GLI reference values have been evaluated and considered applicable for both Caucasian adults and children [7,8]. Similar matters have also been argued previously by Stanojevic et al. [14]. However, despite the fact that the GLI reference values may be applicable for Caucasian populations in several countries, the present findings demonstrate that there are differences between countries that have to be considered.

The OLIN-studies have conducted research about obstructive lung disease in Northern Sweden since 1985 [26] and the research staff carrying out the spirometric measurements are highly experienced. The sampling of the reference population was rigorously thorough, as was the data quality and repeatability control. The reference population originates from randomly selected healthy non-smokers of the general population of Norrbotten, the northernmost province of Sweden. Selection bias such as using health personnel [2], employees within certain industries [1,2,23] or subjects visiting a certain clinic is thus avoided. One of the strengths of this study is that data is contemporary, i.e. collected from 2008 to 2013, and thus possible secular trends in this data set can be ruled out. It has previously been shown that 150 subjects of each sex is a sufficient sample size to make a reliable evaluation of the applicability of reference values for spirometry [24], and hence this evaluation can be considered convincingly reliable. A possible weakness of this study is that no data of cotinine levels were analysed to confirm non-smoking.

This Swedish study showed a positive offset for observed FEV_1_ and FVC compared to the GLI reference values, with mean Z-scores for FEV_1_ and FVC above the expected for both sexes and across almost all ages. Mean values of FEV_1_ and, in particular, FVC as percent of predicted values exceeded 100% to a greater extent among women than among men. In this study, FVC as percent of predicted value exceeded 100% also according to most of the reference values from other areas, i.e. reference values ECSC [1], Hankinson [20], Langhammer [21] and Gulsvik [22]. The GLI reference values yield similar results as Hankinson’s, with percent of predicted values closer to 100% compared to the previously recommended ECSC reference values, in line with results from previous studies [6]. Reference values from Sweden [3,4] and Finland [23] yielded mean percent of predicted values closer to 100%. However, recent debate criticise the use of percent predicted due to the sex-, height- and age-related bias embedded in this measure, and advocates the use of Z-scores instead [6,27].

The standard deviations for FEV_1_ and FVC Z-scores were close to 0.9 for both sexes, implying that the dispersion around the mean was lower in this sample compared to the GLI. Consequently the LLN for these values may be “too low”. Almost 10% of the subjects were outside the 90% limits of normality as defined by GLI (6.4% for FEV_1_, 9.8% for FVC), but most of those subjects were located above the 95th percentile. The authors of the study which evaluated the applicability of GLI on an Australasian population argue that Z-score deviations <0.5 (corresponding to <3% deviations) are clinically insignificant [7]. In this study however, the deviation of 0.42 Z-scores for FVC among women represent a deviation of 6%. The classification into severity grades of airway and lung disease often relies on FEV_1_ or FVC as percent of a reference value, and thus the use of GLI may lead to invalid classification of disease severity in Sweden.

The mean predicted FEV_1_/FVC ratio was higher compared to the mean observed ratio, and more pronounced so among women compared to men. The Z-score SD’s for both sexes were consistently below 0.9 for the ratio, implying a lower variability in this Swedish dataset also for the ratio. Since the spirometric definition of airway obstruction relies on the ratio, the fact that the GLI predicted ratios are higher means that the prevalence of obstruction may be overestimated in Sweden. Additionally, since the dispersion around the ratio is lower in Sweden compared to GLI, use of the GLI LLN criteria may overestimate the prevalence of obstruction even further. LLN will by definition allow for a 1/20 false positive rate, and this study clearly illustrates that among healthy subjects, those identified as obstructive by the LLN criterion in particular are those with high FVC values.

Regardless of criteria for airway obstruction, the prevalence was higher among women than men in the reference population. If the agreement with GLI is perfect, no such sex-difference should exist when applying the LLN criteria of obstruction based on the GLI reference values. In this study, 9.4% of the women were identified as obstructive according to the GLI LLN criterion (fifth percentile), which indicates that this criterion may overestimate the prevalence of airway obstruction in Swedish women.

## Conclusions

In conclusion; the GLI reference values are preferable compared to the ECSC for Swedish adults. However, among non-smoking healthy men and women in northern Sweden, the mean values of FEV_1_ were somewhat larger compared to those in the non-smoking healthy GLI reference population. A greater discrepancy was found for FVC, especially among women. The use of the LLN criterion for airway obstruction based on the GLI reference values for the FEV_1_/FVC ratio may produce biased prevalence estimates of airway obstruction in Sweden, in particular among women. These results demonstrate the importance of validating the GLI reference values in different countries.

## Additional file

Additional file 1:
**Eligibility criteria for the reference population.** Includes a list of eligibility criteria for inclusion in the reference population.

## Abbreviations

GLI: Global Lung Function Initiative
GOLD: Global Initiative for Chronic Obstructive Lung Disease
OLIN: Obstructive Lung Disease in Norrbotten
ERS: European Respiratory Society
ATS: American Thoracic Society
ECSC: European Coal and Steel Community
LLN: Lower Limit of Normal
FEV_1_: Forced Expiratory Volume in one second
FVC: Forced expiratory Vital Capacity
SVC: Slow expiratory Vital Capacity
VC: Vital Capacity (the highest value of FVC and SVC)
