# Supplementary material for: Evaluation of the global lung function initiative 2012 reference values for spirometry in a Swedish population sample
Source: BMC Pulm Med. 2015 Mar 25;15:26. doi: 10.1186/s12890-015-0022-2 (PMC4417328; doi:10.1186/s12890-015-0022-2)
Supplement: Additional file 1: — Eligibility criteria for the reference population. Includes a list of eligibility criteria for inclusion in the reference population. [file 12890_2015_22_MOESM1_ESM.docx]

## Additional file 1

Title: Eligibility criteria for the reference population

| Information collected within standardized interviews during 2008-2013 | Criteria |
| --- | --- |
| Ethnicity | Caucasian |
| Smoking habits | Non-smokers with a maximum of 1 packyear |
| mMRC dyspnea scale* | 0 or 1 |
| Usually wheeze when breathing | No |
| Sputum production when coughing most days in periods of 3 months per year | No |
| Have or ever have had asthma | No |
| Been diagnosed as having asthma by a physician | No |
| Use of asthma medication last 12 months, regularly or when needed | No |
| Previous use of asthma medication | No |
| Been diagnosed as having chronic bronchitis by a physician | No |
| Been diagnosed as having Chronic Obstructive Pulmonary Disease (COPD) by a physician | No |
| Been diagnosed as having emphysema by a physician | No |
| Use of medication for chronic bronchitis, COPD or emphysema last 12 months | No |
| Previous use of medication for chronic bronchitis, COPD or emphysema | No |
| Ischemic heart disease | No |
| Wheeze last 12 months with sputum in the chest | No |
| Wheeze last 12 months with concurrent breathlessness | No |
| Wheeze last 12 months without having a cold | No |
| Wheeze last 12 months most days per week | No |
| Other disability that could affect the lung capacity | No |

*mMRC=modified Medical Research Council
